# Zika virus oncolytic activity requires CD8^+^ T cells and is boosted by immune checkpoint blockade

**DOI:** 10.1172/jci.insight.144619

**Published:** 2021-01-11

**Authors:** Sharmila Nair, Luciano Mazzoccoli, Arijita Jash, Jennifer Govero, Sachendra S. Bais, Tong Hu, Camila R. Fontes-Garfias, Chao Shan, Hideho Okada, Sujan Shresta, Jeremy N. Rich, Pei-Yong Shi, Michael S. Diamond, Milan G. Chheda

**Affiliations:** 1 Department of Medicine, Washington University School of Medicine, St. Louis, Missouri, USA.; 2 Department of Biochemistry and Molecular Biology, University of Texas Medical Branch, Galveston, Texas, USA.; 3 Department of Neurological Surgery and; 4 Parker Institute for Cancer Immunotherapy, University of California San Francisco, San Francisco, California, USA.; 5 Center for Infectious Disease and Vaccine Research, La Jolla Institute for Immunology, La Jolla, California, USA.; 6 Division of Regenerative Medicine, Department of Medicine, and; 7 Department of Neurosciences, University of California School of Medicine, San Diego, La Jolla, California, USA.; 8 Sanford Consortium for Regenerative Medicine, La Jolla, California, USA.; 9 Department of Pharmacology and Toxicology and; 10 Sealy Center for Structural Biology and Molecular Biophysics and Sealy Center for Vaccine Development, University of Texas Medical Branch, Galveston, Texas, USA.; 11 Department of Pathology & Immunology,; 12 Department of Molecular Microbiology,; 13 The Andrew M. and Jane M. Bursky Center for Human Immunology & Immunotherapy Programs, and; 14 Department of Neurology, Washington University School of Medicine, St. Louis, Missouri, USA.

**Keywords:** Immunology, Oncology, Brain cancer, Cancer immunotherapy

## Abstract

Glioblastoma multiforme (GBM) is a fatal human cancer in part because GBM stem cells are resistant to therapy and recurrence is inevitable. Previously, we demonstrated Zika virus (ZIKV) targets GBM stem cells and prevents death of mice with gliomas. Here, we evaluated the immunological basis of ZIKV-mediated protection against GBM. Introduction of ZIKV into the brain tumor increased recruitment of CD8^+^ T and myeloid cells to the tumor microenvironment. CD8^+^ T cells were required for ZIKV-dependent tumor clearance because survival benefits were lost with CD8^+^ T cell depletion. Moreover, while anti–PD-1 antibody monotherapy moderately improved tumor survival, when coadministered with ZIKV, survival increased. ZIKV-mediated tumor clearance also resulted in durable protection against syngeneic tumor rechallenge, which also depended on CD8^+^ T cells. To address safety concerns, we generated an immune-sensitized ZIKV strain, which was effective alone or in combination with immunotherapy. Thus, oncolytic ZIKV treatment can be leveraged by immunotherapies, which may prompt combination treatment paradigms for adult patients with GBM.

## Graphical abstract

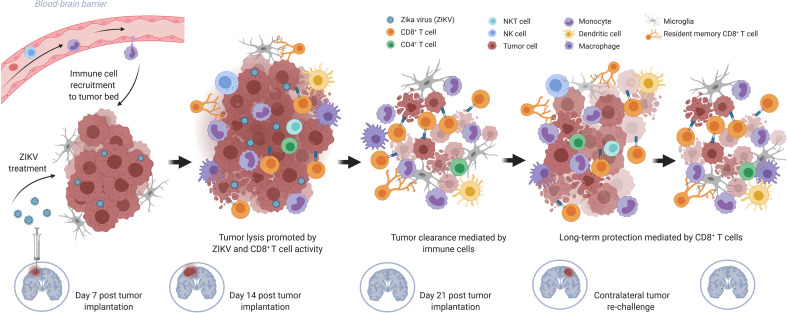

## Introduction

Glioblastoma multiforme (GBM) is the most aggressive primary brain tumor, and virtually all patients die within 2 years of diagnosis (1). Standard treatment includes surgery, radiation, temozolomide chemotherapy, and more recently, adjuvant use of tumor treating fields (2). Despite maximal treatment, most GBMs recur within 6 months, at which time no standard or curative treatment exists. Poor patient outcomes from GBM are multifactorial, including the presence of GBM stem cells (GSCs), which are resistant to radiation treatment and chemotherapy (3–5), and weak antitumor immunological responses (6–11).

We and others demonstrated that Zika virus (ZIKV), a flavivirus that emerged in 2015 as a cause of congenital brain anomalies, has specific lytic activity against GSCs (12–17). GSCs share properties with fetal neuronal progenitor cells (18–22). In vivo studies from independent groups validated that oncolytic ZIKV therapy extends survival of glioma-bearing mice (13, 15, 17, 23). Although ZIKV is a neurotropic virus in fetuses, it rarely infects the brain or causes neurological disease in adults (24). Thus, oncolytic ZIKV could have therapeutic potential in adult patients with GBM.

Oncolytic viral therapies for treating GBM include the measles virus (25–28), poliovirus (29, 30), adenovirus (31, 32), herpesviruses (33–36), myxoma virus (37, 38), and vesicular stomatitis virus (39). Clinical trials with several of these oncolytic viruses have been reported or are under development for treatment of GBM. However, these therapies do not specifically target GSCs.

GBM is resistant to immunotherapy due, in part, to the immunosuppressive tumor microenvironment (40, 41), which is characterized by T cell dysfunction (42, 43), regulatory T cell–imposed tolerance (6, 44, 45), inadequate antigen presentation (46), and immunosuppressive activity of recruited myeloid suppressor cells (8, 9, 47). In other solid tumors, immune checkpoint blockade targeting inhibitory receptors expressed on T cells such as cytotoxic T lymphocyte–associated protein 4 (CTLA-4) or programmed cell death 1 (PD-1) elicits clinical improvement and tumor regression (48–50). However, clinical trials of immune checkpoint blockade in GBM have largely failed (51–55). One small study in recurrent GBM using anti–PD-1 immunotherapy before tumor resection extended median survival by 5 months (56), suggesting the timing of the therapy might be important.

Overcoming resistance to immune checkpoint blockade and augmenting immune responses that suppress tumor growth is a therapeutic priority in GBM. In this context, it is notable that ZIKV treatment reduces tumor size and extends survival in mice beyond that expected for its anti-GSC effects. We hypothesized that oncolytic ZIKV treatment of GBM reshapes the immunological microenvironment, which might be leveraged further by immunotherapy. Here, we evaluate the immunological basis of protection mediated by ZIKV therapy and establish ways to enhance its efficacy.

## Results

### ZIKV increases CD8^+^ T cell infiltration into the tumor bed.

**Figure 1:**
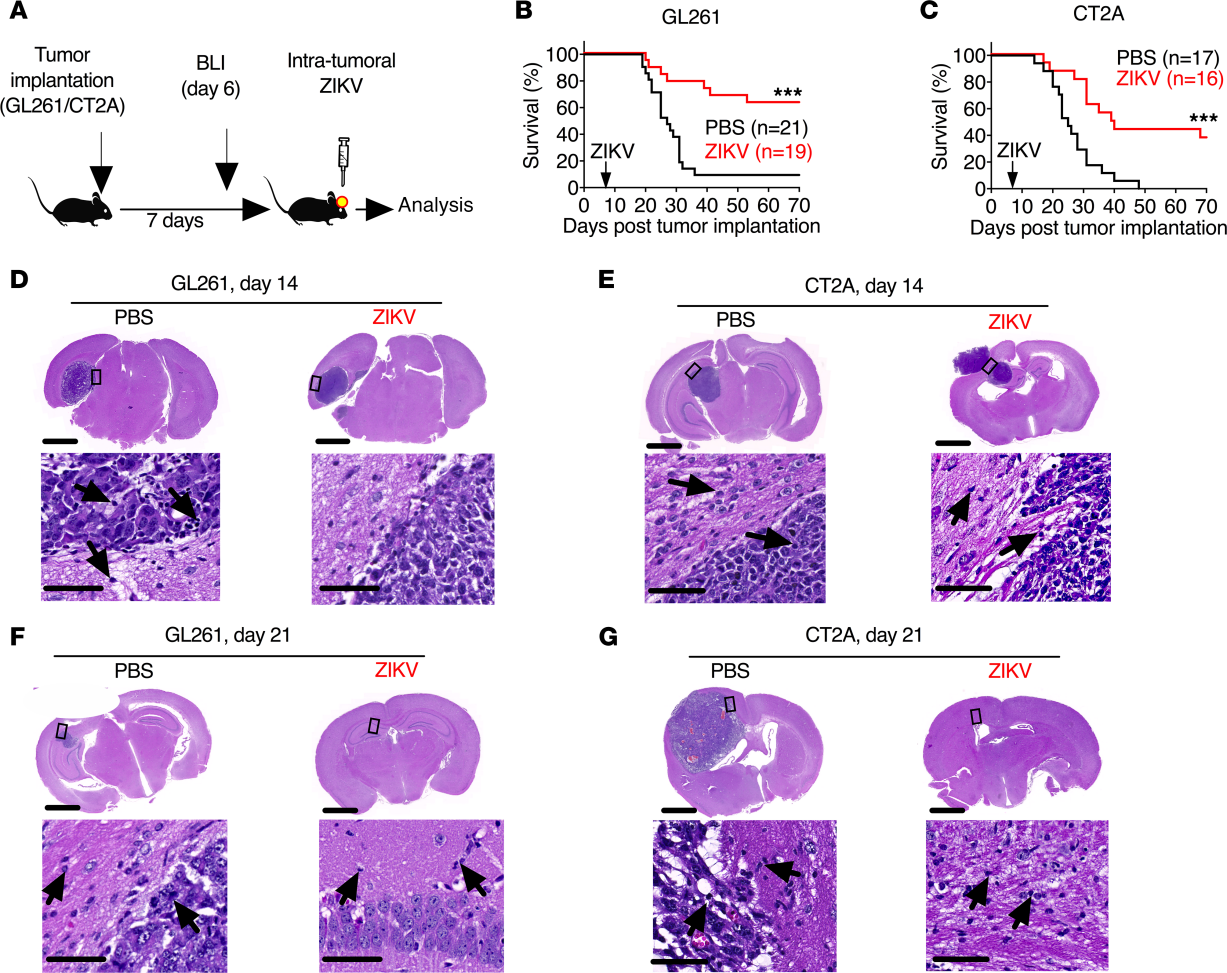
ZIKV extends survival of glioma-bearing mice. (**A**) Scheme of experiments. C57BL6/J mice were implanted with 4 × 10^4^ GL261 or CT2A glioma cells transduced with luciferase and treated intratumorally with 10^5^ FFU mouse-adapted ZIKV-Dakar or PBS control on day 7. (**B** and **C**) Survival analysis of mice bearing GL261 (*n* = 19–21 mice) (**B**) or CT2A (*n* = 16–17) (**C**). (**D**–**G**) Images of hematoxylin and eosin staining of coronal brain sections at 7 and 14 days after ZIKV treatment. Scale bars: 1000 μm (top), 50 μm (bottom). Arrows indicate immune cells. Statistical differences were determined by (**B** and **C**) log-rank test: ***P < 0.001. All data are pooled from at least 2 to 3 independent experiments.

We implanted 2 syngeneic glioma cell models, GL261 and CT2A transduced with a luciferase reporter, by stereotactic injection into the right cerebral hemisphere of 8-week-old C57BL6/J mice. After confirming tumor growth using bioluminescence, we randomized mice to intratumor treatment with either mouse-adapted ZIKV-Dakar strain (10^5^ focus-forming units [FFU]) or PBS (57) (Figure 1A). ZIKV treatment increased median survival, and the long-term survival rates increased from approximately 10% to 63% for GL261-bearing mice and 0% to 37% for CT2A-bearing mice (Figure 1, B and C). Histological analysis revealed comparable tumor sizes between the ZIKV and PBS groups at day 14 after tumor implantation (7 days after ZIKV treatment) but a decrease in tumor size 1 week later at day 21 after tumor implantation (14 days after ZIKV treatment) in response to ZIKV treatment (Figure 1, D and E). We also observed infiltration of immune cells in the tumor microenvironment at days 14 and 21 after tumor implantation in animals treated with ZIKV (7 and 14 days after ZIKV treatment) (Figure 1, F and G). Analysis of the kinetics of viral replication in the brain revealed that ZIKV RNA was cleared by 14 days postinfection (Supplemental Figure 1; supplemental material available online with this article; https://doi.org/10.1172/jci.insight.144619DS1).

**Figure 2:**
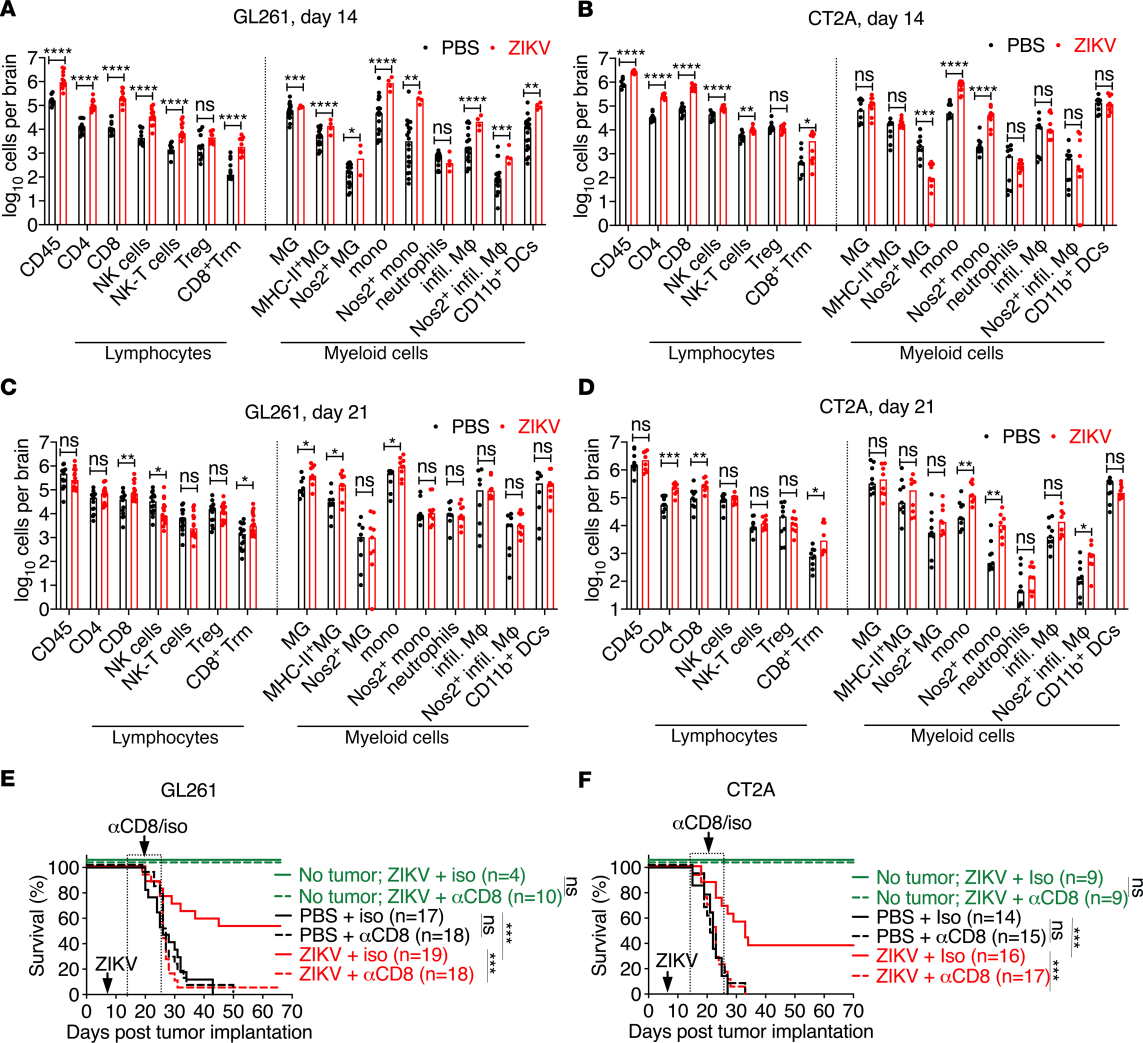
CD8^+^ T cells are required for ZIKV efficacy in mice bearing primary tumors. (**A**–**D**) Absolute numbers of immune cells in the brain at 14 and 21 days after tumor implantation (7 and 14 days after ZIKV treatment). Bars indicate median values. (**E** and **F**) Survival analysis of mice bearing GL261 (*n* = 17–19) (**E**) or CT2A (*n* = 14–17) (**F**) glioma cells, treated with ZIKV or PBS on day 7 and anti-CD8 or isotype control antibody as described in the Methods. Mice without tumor (green lines) (*n* = 9) were similarly treated. Statistical differences were determined by (**A**–**D**) Mann-Whitney *U* test: *P < 0.05, **P < 0.01, ****P < 0.0001; and log-rank test: ***P < 0.001. All data are pooled from at least 2 to 3 independent experiments. MG, microglia.

We quantified the infiltrating immune cell composition (lymphoid and myeloid cells) in the brain at 14 and 21 days after tumor implantation (7 and 14 days after ZIKV treatment) using flow cytometry (Supplemental Figure 2). Analysis of cells at 14 days after GL261 tumor implantation revealed that ZIKV treatment promoted increased numbers of CD45^+^ leukocytes (~6.5-fold), including CD4^+^ T cells (~7.8-fold), CD8^+^ T cells (~20.1-fold), CD3^–^NK1.1^+^ natural killer (NK) cells (~8-fold), CD3^+^NK1.1^+^ NKT cells (~4.8-fold), and CD8^+^CD44^+^CD69^+^CD103^+^ resident memory T cells (Trm) (~14.6-fold) compared with PBS-treated, tumor-bearing mice (Figure 2A). Similarly, in the CT2A tumor model, ZIKV treatment elicited an increase in numbers of CD45^+^ leukocytes (~4-fold), including CD4^+^ T cells (~7.5-fold), CD8^+^ T cells (~8.9-fold), NK cells (~2-fold), NKT cells (~2-fold), and Trm cells (~8-fold), compared with control-treated mice (Figure 2B). The numbers of FoxP3^+^ regulatory T cells (Tregs) were similar between ZIKV-treated and PBS-treated controls in both the GL261 and CT2A models. By 21 days after GL261 or CT2A tumor implantation (14 days after ZIKV treatment), animals treated with ZIKV treatment had increased numbers of CD8^+^ T cells (~2- and 2.8-fold, respectively) and CD8^+^ memory T cells (~2- and 3.8-fold, respectively) whereas we detected no differences in numbers of CD4^+^ T cells, NKT cells, and Tregs. We observed a reduction of NK cells (~4-fold) at this point in ZIKV-treated animals (Figure 2, C and D). Comparison of immune cells from GL261 tumor–bearing mice to infection with ZIKV alone (no tumor) revealed that ZIKV generated a greater CD8^+^ T response than the tumor itself (~2.2-fold), whereas tumors were associated with greater numbers of NK cells than ZIKV alone (~4.5-fold) at day 21 after tumor implantation or 14 days after ZIKV treatment (Supplemental Figure 3).

A similar analysis of lymphoid cells at 21 days after CT2A tumor implantation revealed no difference in numbers of NK cells, NKT cells, or Tregs but an increase in numbers of CD4^+^ T cells (~4.2-fold) (Figure 2D). Collectively, the data from the 2 glioma models suggest that ZIKV treatment in glioma-bearing mice results in enhanced infiltration of multiple lymphoid cell subsets. While the differences in the early recruitment of immune cells resolved for most cell types, by later points ZIKV-treated gliomas sustained increased numbers of CD8^+^ T cells and CD8^+^ Trm in the tumor bed.

Given that the immune-suppressive tumor microenvironment in gliomas downregulates major histocompatibility complex (MHC) antigen expression and compromises the ability of myeloid cells to cross-present antigen to cytotoxic T cells (46, 58, 59), we hypothesized that ZIKV treatment of gliomas might trigger inflammatory responses that activate microglia and recruit antigen-presenting cells into the tumor region. To evaluate this idea, we analyzed myeloid cells and their activation state in the brain in response to ZIKV treatment in GL261 glioma– or CT2A glioma–bearing mice. At 14 days after GL261 tumor implantation (7 days after ZIKV treatment), ZIKV treatment was associated with a small increase in total numbers of microglia (~1.8 fold) but a more substantial increase in numbers of MHC class II–expressing microglia (~4-fold) (Figure 2A). ZIKV treatment of GL261 gliomas also resulted in increased recruitment of Ly6C^+^ monocytes (31-fold), F4/80^+^ macrophages (~15-fold), and CD11b^+^ monocyte-derived DCs (~8-fold) (Figure 2A). Also seen were increased numbers of inducible NOS–producing microglia (~4.3-fold), monocytes (~29-fold), and macrophages (~7.5-fold), suggesting an enhanced inflammatory potential of myeloid cells in the tumor bed (Figure 2A). ZIKV treatment of both GL261 and CT2A tumor models had limited effects on neutrophil recruitment to the tumor bed (Figure 2, A and B). Whereas ZIKV treatment of CT2A glioma also led to rapid increases in the numbers of Ly6C^+^ monocytes (~16.5-fold) and inducible NOS–producing Ly6C^+^ monocytes (~24-fold) in the brain by day 14, it was not associated with activation of microglia, F4/80^+^ macrophages, or CD11b^+^ monocyte-derived DCs (Figure 2B). At 21 days after GL261 tumor implantation (14 days after ZIKV treatment), we observed increased numbers of microglia (~3.8 fold) and MHC class II–expressing microglia (~5.2-fold) (Figure 2C). However, these differences at day 21 were not detected in the ZIKV-treated, CT2A tumor–bearing mice or tumor-naive, PBS-treated counterparts (Figure 2, C and D). At 21 days after GL261 and CT2A tumor implantation (14 days after ZIKV treatment), continued increases in numbers of Ly6C^+^ monocytes (~2- and 6.8-fold, respectively) were observed but not in numbers of neutrophils, F4/80^+^ macrophages, or CD11b^+^ monocyte-derived DCs (Figure 2, C and D).

### CD8^+^ T cells are required for ZIKV-mediated glioma clearance.

As we observed consistent increases in CD8^+^ T cells in the brain following ZIKV treatment of glioma, we hypothesized that these cells contribute to tumor clearance. To investigate this idea, we performed CD8^+^ T cell depletion studies. Beginning 14 days after GL261 or CT2A tumor implantation (7 days after ZIKV treatment), we administered a CD8^+^ T cell–depleting or isotype control antibody. Depletion of CD8^+^ T cells abrogated the therapeutic effect of ZIKV in both glioma models (Figure 2, E and F). The loss of efficacy was not due to inadequate control of ZIKV because non–tumor-bearing mice treated with ZIKV survived the CD8^+^ T cell depletion regimen (Figure 2, E and F, dashed green line). Exposure to ZIKV alone was not sufficient to protect mice from subsequent glioma because ZIKV infection before tumor implantation did not confer any survival benefit against tumor (Supplemental Figure 4). Collectively, these data demonstrate the potential importance of ZIKV-instructed CD8^+^ T cells for protection against primary tumor pathogenesis.

### Long-term survivors of glioma after ZIKV treatment are protected against secondary syngeneic glioma in a CD8^+^ T cell–dependent manner.

**Figure 3:**
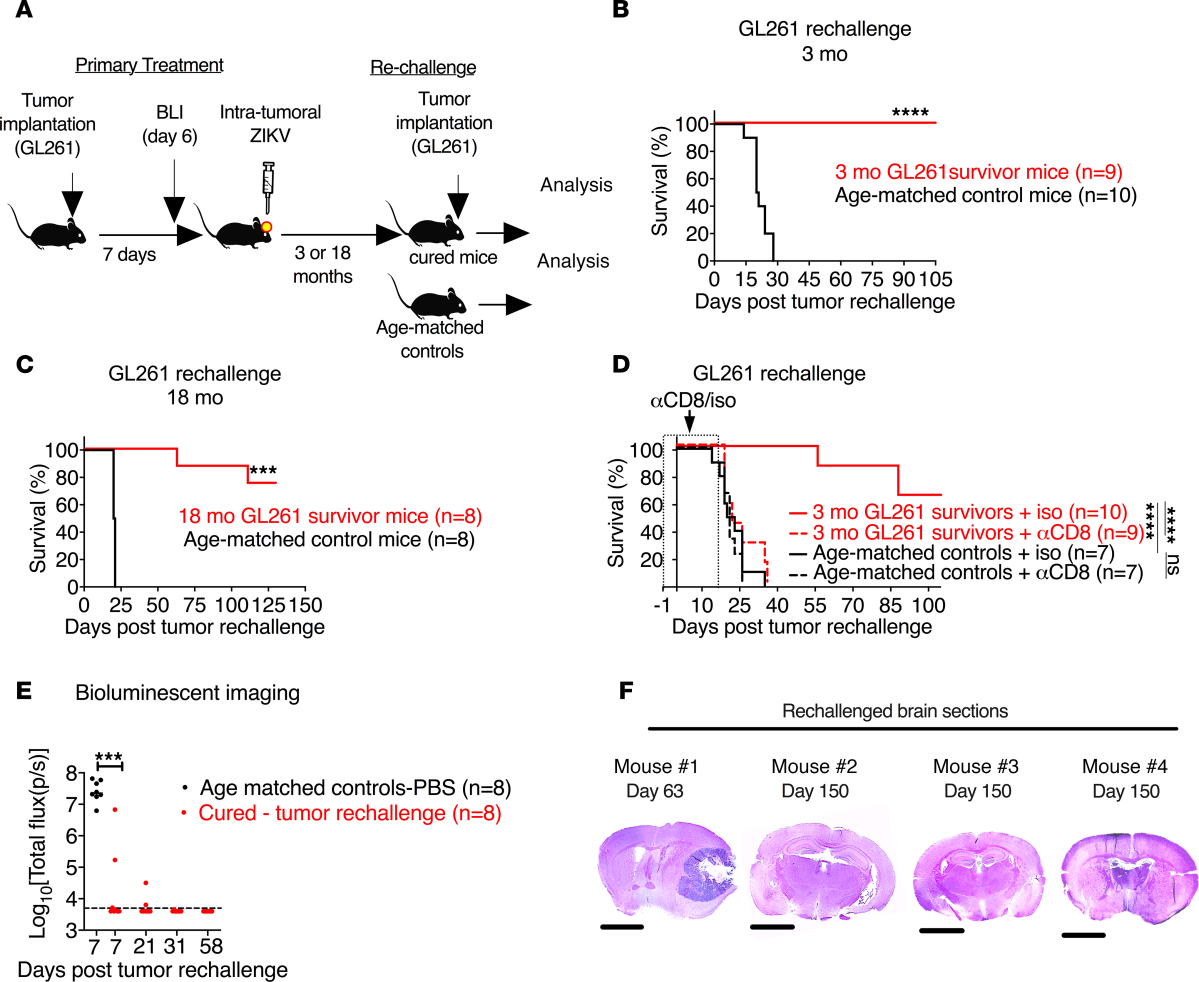
CD8^+^ T cells are required for ZIKV efficacy in mice during rechallenge. (**A**) Scheme of tumor rechallenge experiments. (**B** and **C**) Surviving mice from GL261 studies with ZIKV were rechallenged 3 months (**B**) (*n* = 9–10) or 18 months later (**C**) (*n* = 8) with 4 × 10^4^ GL261 cells on the contralateral side. Age-matched (20 months old, *n* = 10; and 26 months old, *n* = 8) naive mice served as controls. (**D**) Surviving mice from GL261 studies with ZIKV were rechallenged 3 months later with 4 × 10^4^ GL261 cells and treated with antibodies against CD8 or isotype control as described in the Methods. Age-matched (20 months old; *n* = 7) mice served as controls. (**E**) Photon flux (photons/s) of bioluminescent images of brains of mice described from **C** at indicated times after rechallenge. (**F**) Representative images from **C** of hematoxylin and eosin staining of coronal brain sections from a mouse that did not survive rechallenge (mouse 1) and those surviving up to day 150 after rechallenge (mice 2–4). Scale bars represent 1000 μm. Horizontal lines indicate median values. The dotted line denotes the limit of detection (**E**). Statistical differences were determined by (**B**–**D**) log-rank test: ***P < 0.001, ****P < 0.0001; and (**E**) Mann-Whitney *U* test: ***P < 0.001. Data are pooled from at least 2 independent experiments.

To model the tumor recurrence that occurs inevitably in patients, we performed rechallenge experiments in mice that were long-term survivors of GL261 gliomas after ZIKV treatment. We implanted syngeneic GL261 cells into the contralateral side of the brains of ZIKV-treated tumor survivors, 3 months or even 1.5 years after primary tumor implantation (Figure 3A). Whereas age-matched tumor-naive mice succumbed to the GL261 tumor as expected, ZIKV-treated tumor survivors were protected against syngeneic tumor rechallenge and survived for at least 150 days (Figure 3, B and C). We next evaluated whether memory CD8^+^ T cell responses after ZIKV treatment prevent growth of the secondary syngeneic tumor. When we depleted CD8^+^ T cells before GL261 tumor rechallenge, protective phenotype was reversed (Figure 3D). To understand the temporal dynamics of tumor formation following rechallenge, we performed serial bioluminescence imaging of mice that were rechallenged 18 months after their treatment with ZIKV. Age-matched control mice had luciferase signals at day 7, and they succumbed to tumor by day 21. Although 2 of the rechallenged mice had luciferase signals at day 7 after rechallenge, they had little to no luciferase signals by day 58, suggesting tumors either did not engraft or did not grow in these mice (Figure 3E). Histological analysis of survivors at 150 days after tumor rechallenge revealed no evidence of tumor (Figure 3F). In contrast, the 1 mouse that did not survive the rechallenge had luciferase signal above the limit of detection at days 7 and 21 and had extensive tumor at day 63 after rechallenge (Figure 3, E and F). Analysis of rechallenged mice that died beyond day 100 did not show any signs of tumor formation (data not shown), suggesting they likely died from nontumor causes, as they were 2.5 years old and near the end of their natural life span. Collectively, these data demonstrate the potential importance of ZIKV-instructed memory CD8^+^ T cells for protection against secondary tumor development.

### ZIKV treatment improves the response to immune checkpoint blockade.

**Figure 4:**
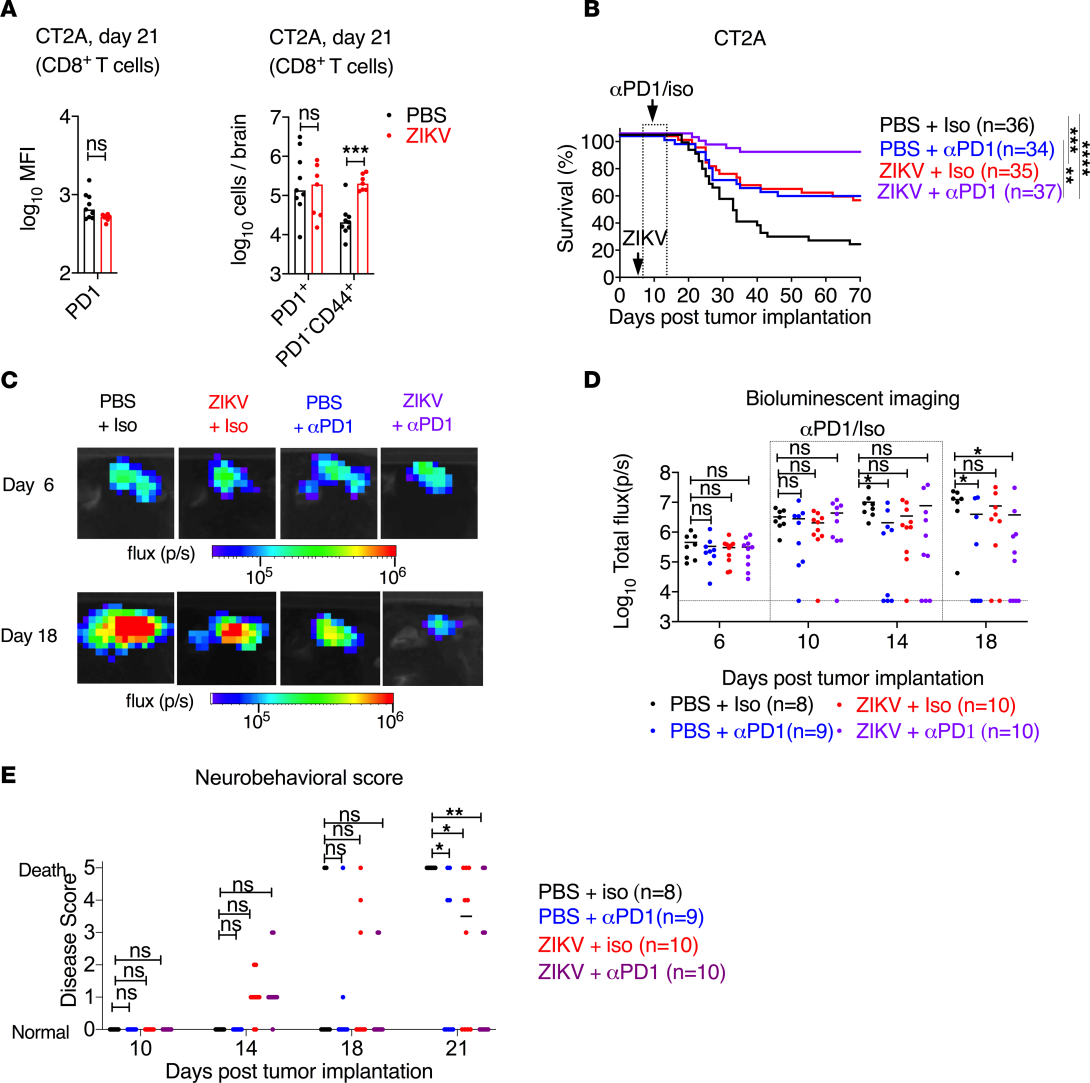
ZIKV and anti–PD-1 protect against glioma in mice. (**A**) MFI of PD-1 expression on CD8^+^ T cells and total numbers of PD1^+^CD8^+^ T cells and PD1^–^CD8^+^CD44^+^ T cells from PBS- or ZIKV-treated, glioma-bearing mice at day 21 after tumor implantation (14 days after ZIKV treatment). (**B**) Survival analysis of mice bearing CT2A tumors, treated with ZIKV or PBS on day 7 and anti–PD-1 or isotype control antibody as described in the Methods (*n* = 34–37). (**C**) Representative images from **B** at day 6 and day 18 after tumor implantation (11 days after ZIKV treatment). (**D**) Photon flux of bioluminescence images from CT2A tumor–bearing mice treated as in **B**. (**E**) Neurobehavioral score (0 to 5) as described in Methods in CT2A tumor–bearing mice treated with antibody against PD-1 or isotype control. Bars/horizontal lines indicate median values. The dotted line denotes the limit of detection (**D**). Data are from 2 independent experiments. Statistical differences were determined by (**A**) Mann-Whitney *U* test (***P < 0.001), (**B**) log-rank test (**P < 0.01; ***P < 0.001; ****P < 0.0001), and (**D** and **E**) 2-way ANOVA test with Dunnett’s posttest (*P < 0.05; **P < 0.01).

T cell deficits that occur during GBM pathogenesis are characterized by increased expression of immune checkpoint molecules (e.g., PD-1, Tim3, and Lag3) that negatively regulate tumor immune responses (11, 60–63). In fact, GBM infiltrating lymphocytes upregulate PD-1 expression on up to 95% of CD8^+^ T cells (60). We investigated whether ZIKV infection changed the expression of immune checkpoint molecules. Flow cytometry analysis revealed that ZIKV treatment did not alter the expression of PD-1, Tim3, or Lag3 or the numbers of PD1^+^, Lag3^+^, or Tim3^+^ CD8^+^ T cells (Figure 4A; and Supplemental Figure 5, A and B). However, the numbers of activated PD1^–^CD44^+^CD8^+^ T cells were higher (~10-fold) in the brains of ZIKV-treated gliomas compared with those treated with PBS control (Figure 3A).

The presence of spontaneous tumor-infiltrating lymphocytes correlates with better prognosis, especially for tumor immunotherapies (64). Because the GBM tumor microenvironment has few T cells, augmenting CD8^+^ T cells numbers is one way to alleviate resistance to immune checkpoint blockade therapy (7). Since ZIKV treatment increases lymphocyte number in the tumor bed, we evaluated whether the combination of ZIKV treatment with checkpoint blockade immunotherapy enhances glioma clearance and promotes survival. We used the CT2A model, which is resistant to immunotherapy (65), and treated tumor-bearing mice with either ZIKV or PBS, with or without anti–PD-1 and its respective isotype control. Combined blockade of PD-1 and ZIKV treatment was superior to either treatment alone (Figure 4B). Serial bioluminescence imaging every 4 days demonstrated that ZIKV- and anti–PD-1–treated tumors regressed at approximately day 18 after tumor implantation (11 days after virus treatment) (Figure 4, C and D). Neurobehavioral disease assessments (0, no disease; 1, ruffled fur, piloerection, weight loss, or slow movements; 2, lethargy, unsteady gait, or hunched back; 3, decreased strength in forelimbs and/or hind limbs; 4, restricted movement, extreme body weight loss (over 20%), or convulsions; and 5, moribund or death) revealed fewer central nervous system deficits in animals receiving the combination of ZIKV and anti–PD-1 than those with either individual treatment (Figure 4E). However, approximately 15% of mice were resistant to combination therapy and succumbed to tumor burden (Figure 4B).

### An attenuated ZIKV strain with therapeutic potential.

**Figure 5:**
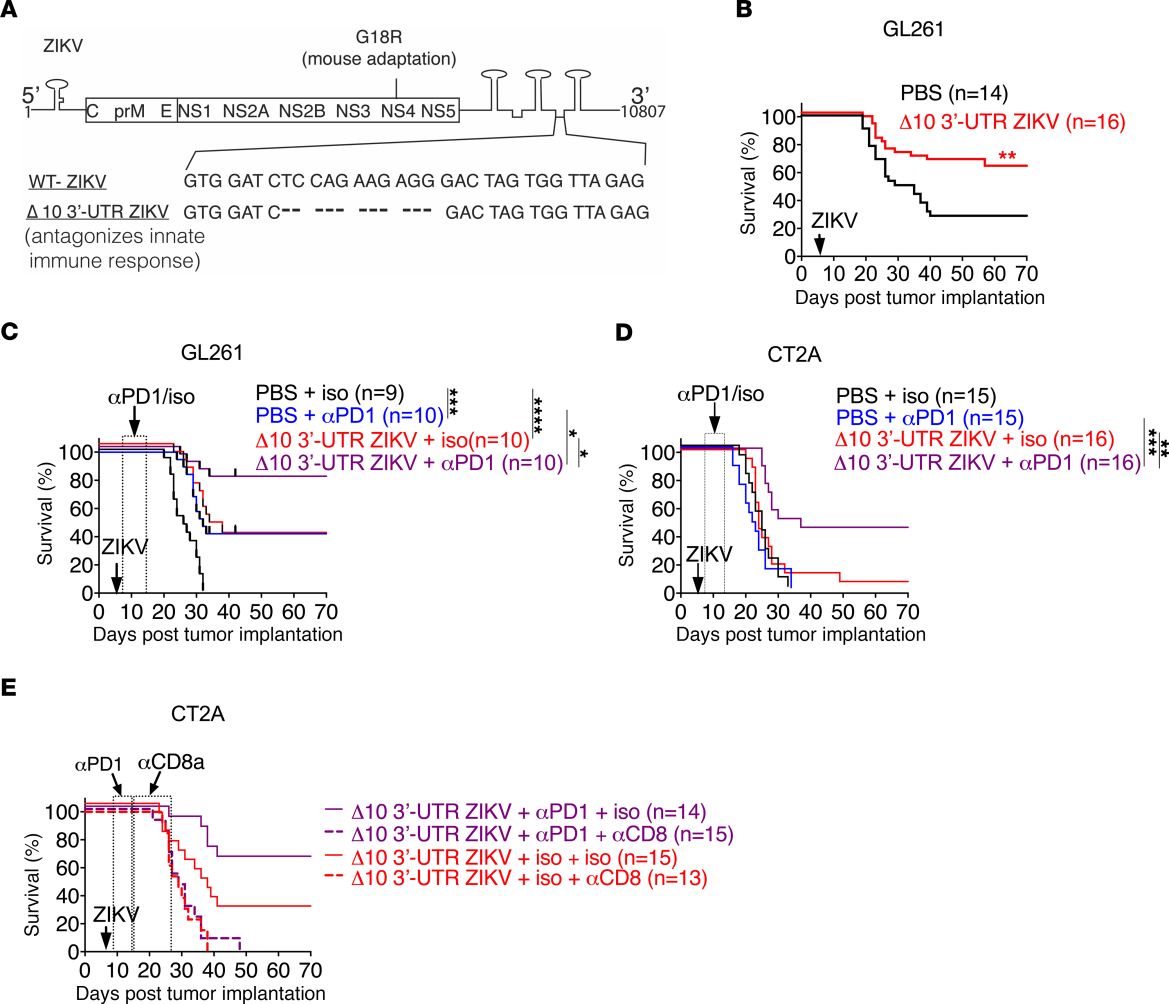
Immune-sensitized Δ10 3′-UTR ZIKV is effective alone or in combination with anti–PD-1 therapy. (**A**) Schematic of Δ10 3′-UTR ZIKV. (**B**) Mice were implanted with GL261 (*n* = 14–16) and treated with 10^6^ FFU of Δ10 3′-UTR ZIKV or PBS on day 7 (downward arrow). (**C** and **D**) Treatment included Δ10 3′-UTR ZIKV, or PBS as in **B**, combined with anti–PD-1 or isotype control antibodies administered days 8, 10, 12, and 14, in mice bearing GL261 (*n* = 9–10) (**C**) or CT2A (*n* = 15–16) (**D**). (**E**) Survival analysis of mice bearing CT2A glioma cells, treated with Δ10 3′-UTR ZIKV and anti–PD-1 or isotype control antibody as well as anti-CD8 or isotype control antibody as described in the Methods (*n* = 13–15). Data are pooled from 2 independent experiments. Statistical differences were determined by the log-rank test (**P < 0.01; ***P < 0.001).

Although there are few reports of ZIKV-induced encephalitis in adults (66, 67), safety is paramount in any potential clinical application. To further develop ZIKV as an oncolytic therapy, we designed a safer strain by making it more sensitive to the host innate immune response without compromising its ability to replicate in and kill GSCs. We engineered a deletion of 10 nucleotides in the 3′ untranslated region (Δ10 3′-UTR ZIKV) of the ZIKV-Dakar cDNA clone (Figure 5A). This deletion abrogates production of a short subgenomic flaviviral RNA species that antagonizes cell-intrinsic innate immune responses (15, 68). The Δ10 3′-UTR ZIKV is attenuated in immunocompromised mice, and this mutation also is the basis of a candidate ZIKV vaccine (68). We compared the tumoricidal effects of the parental virus and Δ10 3′-UTR ZIKV against mouse glioma cell lines (CT2A, GL261, SB28) and human GSCs (0308, 667, 3565, 387) (Supplemental Figure 6). The Δ10 3′-UTR ZIKV strain displayed anti-GSC oncolytic activity that was similar to the parental virus strain. In vivo, this immune-sensitized Δ10 3′-UTR ZIKV also retained efficacy, as treatment increased the survival rate of GL261 tumor–bearing mice (Figure 5B). We also determined whether treatment of Δ10 3′-UTR ZIKV in combination with anti–PD-1 immunotherapy enhanced survival, as it did for the parent WT-ZIKV strain. We treated GL261 and CT2A tumor–bearing mice with either Δ10 3′-UTR ZIKV or PBS, with or without anti–PD-1 or isotype control mAbs. Whereas Δ10 3′-UTR ZIKV or anti–PD-1 therapy individual treatments improved long-term survival rates in GL261 tumor–bearing mice from 0% to 32% (anti–PD-1 alone) and 33% (Δ10 3′-UTR ZIKV alone), combination therapy improved long-term survival to approximately 80% (Figure 5C). In the CT2A tumor model, the median survival times of Δ10 3′-UTR ZIKV, anti–PD-1, and PBS treatment with isotype controls were 24 days, 25 days, and 22 days, respectively (Figure 5D), suggesting little benefit of immune-sensitized virus treatment alone on survival of these glioma-bearing mice. However, the combination of 10 3′-UTR ZIKV with anti–PD-1 prolonged median survival to 33.5 days after tumor implantation, and the survivor rate increased from 0% to approximately 40% in the combination treatment group (Figure 5D). CD8 depletion of the Δ10 3′-UTR ZIKV and anti–PD-1 combination treatment group reversed the phenotype, suggesting that the efficacy was driven by CD8^+^ T cells (Figure 5E). Thus, our data demonstrate that combined Δ10 3′-UTR ZIKV treatment and PD-1 blockade had efficacy, and this was better than either regimen alone.

## Discussion

GBM remains a clinical challenge. Despite the advances in cataloging tumor genomic alterations through large-scale projects like The Cancer Genome Atlas, precision medicine has not yet changed dismal patient outcomes (15). A major barrier has been intratumor heterogeneity, and immunotherapy and oncolytic treatment provide the opportunity to destroy transformed cells across a diverse tumor genetic landscape (15, 69). Despite the success of immunotherapies for solid tumors, such as melanoma (48, 70) and non–small cell lung cancer (71), the treatments have largely failed in GBM (50–54). GBM harbors a low mutational burden and exerts a potent immunosuppressive effect on the microenvironment (72). Also relevant is the correlation between the cancer stem cell frequency in tumors and weakness of the antitumor immune response (73). Thus, a central problem in GBM remains finding ways to induce a robust immunological response against the tumor. Here we show that ZIKV treatment remodels the GBM microenvironment and supports a CD8^+^ T cell infiltrative response in the tumor environment, and this is crucial for therapeutic efficacy against both primary and secondary tumors. Moreover, treatment using a parental or immune-sensitized, attenuated ZIKV strain converts the poorly inflamed tumor environment into an immunostimulatory one that overcomes resistance to anti–PD-1 treatment. Further studies are needed to clarify whether ZIKV treatment improves functional antitumor CD8^+^ T cell responses against GSCs or common glioma antigens by promoting antigen cross-presentation or other immunomodulatory mechanisms.

Oncolytic viral therapy for solid tumors has been successful in cancer; the recent FDA approval of talimogene laherparepvec (T-VEC), a genetically engineered herpesvirus to treat melanoma, was a milestone. Investigating viral agents in brain tumors is not new; over the last 3 decades, there have been a number of attempts to use viruses as either gene therapy delivery systems or as oncolytic agents. In the early 1990s, Martuza and colleagues engineered herpes simplex virus capable of selective replication and killing of GBM (36). Since that time, oncolytic viruses have been shown to target GBM in multiple ways: direct tumor killing combined with activation of innate and antitumor T cell responses. Oncolytic strains of herpesvirus (G47Δ) (34), measles virus (MV-141.7/MV-AC133) (26), adenovirus virus (DNX2401 in ClinicalTrials.gov NCT03714334 NCT02197169, and NCT01956734 or AdFlt3L/AdTK) (74–76), myxoma virus (37), vaccinia virus (NCT03294486) (77), and poliovirus (PVSRIPO, NCT04479241) (29) are now under evaluation in GBM. The differential properties and relative advantages of each of these viruses, and even ZIKV, remain poorly understood. One potential advantage of ZIKV is its specificity against GSCs, a highly treatment-resistant subpopulation of GBM cells that may drive recurrence. Our results suggest that the efficacy of ZIKV stems from GSC targeting and its ability to induce immune responses that facilitate CD8^+^ T cell–dependent clearance of tumor components not directly killed by ZIKV.

Solid tumors with low amounts of T cell infiltration generally do not benefit from immune checkpoint blockade therapy (78–80). In these cases, oncolytic virotherapy is an attractive treatment option, as virus-induced inflammation can enhance efficacy of checkpoint blockade therapy. A study in humans showed that T-VEC with anti–PD-1 immunotherapy in melanoma had a tolerable safety profile, and the combination appeared to have greater efficacy against melanoma than T-VEC or checkpoint blockade monotherapy (81). Due to the success of immune checkpoint inhibitors in other cancers and their possible additive effects with oncolytic viruses, many virus/antibody combinations are currently being investigated in clinical trials (82, 83). This includes an ongoing phase II clinical trial with an oncolytic adenovirus (DNX-2401) combined with pembrolizumab (anti–PD-1) for patients with recurrent GBM (ClinicalTrials.gov NCT02798406). Our data suggest an analogous combination with ZIKV may also be worth pursuing. In addition, future studies in nonresponders to ZIKV and PD-1 blockade combination therapy might identify mechanisms of resistance, such as loss of tumor antigens, reduction of immune infiltration surrounding the tumor, or other mechanisms of T cell anergy or exhaustion.

We observed that ZIKV treatment also increased the tumor-associated myeloid cell response in the tumor bed, particularly the monocyte and microglia populations. Given that tumor-associated macrophage subsets may contribute to antigen presentation and the antitumor immune cycle, or promote tumor cell growth and suppress an immune response (84–86), further studies must clarify what rebalancing and myeloid cell skewing ZIKV treatment initiates.

While previous work has demonstrated that ZIKV replication is largely self-limited to GSCs, partially because of their inherently attenuated innate immune response (13) and expression of key integrin signaling molecules that facilitate infection (14), safety remains a paramount concern. The safer, immune-sensitized strain was less potent in the CT2A glioma model but had significant additive effect with immune checkpoint blockade (Figure 5). However, further histological analysis of the subventricular zone and hippocampus of long-term survivors is required to ensure that normal stem cell niches remain intact from ZIKV treatment. Genetic modifications of Δ10 3′-UTR ZIKV to express cytokines or chemokines, for example, IL-6 (87), IL-12 (88, 89), and/or TNF-α (90, 91), to help manipulate the tumor microenvironment may boost its efficacy (92, 93). Optimization of the timing of ZIKV administration with respect to radiation and chemotherapy, both of which immunosuppress patients, will be important considerations for evaluating its possible clinical use and benefit. Nonetheless, given its unique tropism for GSCs and its combinatorial effects with immune checkpoint blockade, ZIKV offers a potential therapeutic opportunity for adult patients with GBM.

## Methods

### Tumor implantations.

Single-cell suspensions of GL261 or CT2A cells (4 × 10^4^ cells in 4 μL) were implanted into the right cerebral hemisphere of 8- to 9-week-old C57BL6/J female mice (000664, The Jackson Laboratory) after mice were anesthetized with ketamine (10 mg/kg), xylazine (100 mg/kg), and buprenorphine SR (1 μg/g). Mice were mounted onto a stereotactic apparatus (Stoelting), and an incision was made over the cranial midline. A burr hole was made 2.5 mm lateral and 1.5 mm anterior to lambda. A 29.5-gauge Hamilton syringe was inserted to a depth of 3 mm and withdrawn 0.5 mm to a depth of 2.5 mm. The cell suspension was injected over the course of 5 minutes, and the syringe was slowly withdrawn. The incision site was closed by surgical sutures.

### Bioluminescence imaging.

Animals were monitored for tumor development via bioluminescence imaging. Beginning at day 6 after tumor implantation, mice were anesthetized by isoflurane (2% vaporized in oxygen) and were injected intraperitoneally with d-Luciferin (150 mg/kg; Gold Bio) and imaged using an IVS50 imaging system (PerkinElmer). Total photon flux (photons/s) from the tumor was measured using Living Image 2.6 software (PerkinElmer).

### Treatment and animal monitoring.

At day 7 after tumor implantation, mice with similar flux were randomized between groups. Using the same coordinates as for tumor implantation, mice were inoculated intratumorally with mouse-adapted ZIKV (10^5^ FFU), Δ10 3′-UTR-ZIKV (10^6^ FFU), or PBS, each in 10 μL. We injected CD8-depleting antibodies (clone 2.43, Bio X Cell) or isotype control IgG2b (clone LTF-2, Bio X Cell) intraperitoneally starting at day 14 after tumor implantation with an initial dose of 25 mg/kg and followed with booster doses of 12.5 mg/kg every 5 days until day 26. Representative mice were bled to confirm depletion. Checkpoint blockade antibodies against PD-1 (clone 29F.1A12, Bio X Cell), or corresponding IgG2a control (2A3, Bio X Cell), were injected intraperitoneally on days 8, 10, 12, and 14 with a dose of 10 mg/kg. Mice were monitored daily for signs of neurological impairment and were euthanized when moribund. Animal caretakers were blinded to treatments.

### Neurobehavioral score.

Tumor-bearing animals were scored from 0 to 5 based on the following scale: 0, no disease; 1, ruffled fur, piloerection, weight loss, or slow movements; 2, lethargy, unsteady gait, or hunched back; 3, decreased strength in forelimbs and/or hind limbs; 4, restricted movement, extreme body weight loss (over 20%), or convulsions; and 5, moribund or death (94, 95).

### Cells.

Murine glioma cell lines (GL261 and CT2A, H-2^b^) from the laboratory of Yancey Gillespie, University of Alabama at Birmingham, Birmingham, Alabama, USA (13), and SB28 cells generated in-house (96) transduced with luciferase were cultured in DMEM (Invitrogen, Thermo Fisher Scientific) supplemented with 10% FBS (MilliporeSigma) and 1% penicillin G-streptomycin sulfate amphotericin B complex (Corning) at 37°C in an incubator with 5% humidified CO_2_. Cells were dissociated with 0.25% trypsin and 0.53 mM EDTA (Corning). Cells passaged fewer than 5 times were used for all experiments after ensuring their mycoplasma-free status by PCR (Genome Technology Access Center, Washington University).

Human GSCs (lines 0308 from the laboratory of Howard A. Fine, National Cancer Institute, NIH, Bethesda, Maryland, USA; 667 from the laboratory of Cameron W. Brennan, Memorial Sloan Kettering Cancer Center, New York, New York, USA; and 387 and 3565 generated in-hosue) (13, 14, 97, 98) were grown as neurospheres in NBE medium composed of Neurobasal-A medium (Thermo Fisher Scientific), Glutamax 100X (Thermo Fisher Scientific), N2 (100X) (Thermo Fisher Scientific), B27 (50X) supplement without vitamin A (Thermo Fisher Scientific), and recombinant human basic fibroblast growth factor, and epidermal growth factor (200 μg/mL each; PeproTech) and maintained at 37°C with 5% CO_2_. For dissociation, cells were harvested by Accumax cell dissociation reagent (Innovative Cell Technologies).

### In vitro viral infection and GSC proliferation assay.

Cells were plated at 10^3^ cells per well in 96-well plates and allowed to attach and grow overnight. Relative cell number was approximated using CellTiter-Glo (Promega). Cells were inoculated with mouse-adapted ZIKV or Δ10 3′-UTR-ZIKV at a multiplicity of infection (MOI) of 5, and luminescence was measured at 0, 3, 5, and 7 days after infection (Biotek).

### Generation of Δ10 3′-UTR-ZIKV.

Using a recombinant NS4B(G18R) mouse-adapted infectious Dakar 41525 ZIKV cDNA clone (GenBank: KU955591.1, Senegal, 1984), we engineered a 10-nucleotide deletion in the 3′ untranslated region (Δ3′-UTR) as described (68). Constructs were verified by DNA sequencing, and Δ10 3′-UTR-ZIKV was propagated as described (68). In brief, 10-nucleotide deletion ZIKV RNA was in vitro–transcribed using a T7 mMessage mMachine kit (Ambion) from cDNA plasmids prelinearized by ClaI. The RNA was precipitated with lithium chloride, washed with 70% ethanol, resuspended in RNase-free water, quantitated by spectrophotometry, and stored at –80°C in aliquots. The RNA transcripts (10 μg) were electroporated into Vero cells (CCL-81, ATCC) following a previously described protocol (99). The virus derived from RNA transfection, defined as the P0 stock, was propagated in Vero cells as described (57) after inoculating at an MOI of 0.01 and incubating for 72 hours. Viral titers were quantified by plaque assay (100), and the viral genome was confirmed with sequencing.

### Generation of viral stocks.

Both mouse-adapted ZIKV (Dakar strain) (57) and Δ10 3′-UTR-ZIKV viral stocks were propagated in Vero cells after inoculating at an MOI of 0.01 and incubating for 72 hours. Viral titers were quantified by plaque assay (100, 101) and stored at –80°C.

### Plaque assay.

ZIKV-treated, tumor-bearing mice were euthanized on day 7 or day 14 after viral treatment (day 14 or day 21 after tumor implantation). Tissues were stored in −80°C until virus titration. Samples were thawed, weighed, and homogenized with zirconia/silica beads (BioSpec Products) in a MagNA Lyser instrument (Roche Life Science) in 1 mL of infection media, DMEM supplemented with 2% FBS and 1% penicillin G-streptomycin sulfate amphotericin B complex. Samples were clarified by centrifugation (2000*g* at 4°C for 10 minutes), then diluted serially before infection of Vero cells. Plaque assays were overlaid with 1% methylcellulose and 5 days later were fixed with 10% formaldehyde and stained with crystal violet (101).

### Flow cytometry.

Mice were anesthetized with ketamine (10 mg/kg) and xylazine (100 mg/kg), then perfused with 20 mL of 1× PBS (Gibco, Thermo Fisher Scientific). Brains were excised, treated with digestion buffer containing HBSS (Cellgro 21-022-CM), 0.05% Collagenase D (50 mg/mL; MilliporeSigma C-0130), 10 μg/mL DNase I (MilliporeSigma D5025 150KU), 0.1 μg/mL TLCK trypsin inhibitor (MilliporeSigma T-7254), and 10 mM of HEPES (1M; Cellgro 25-060-Cl) at room temperature for 25 minutes, minced, and strained through a 70 μm strainer. Cell suspensions were washed and subjected to gradient centrifugation (1200*g*, 30 minutes, 4°C) in freshly prepared 30% isotonic Percoll (GE Heathcare 17-5445-02) gradient in RPMI (Gibco, Thermo Fisher Scientific). After discarding myelin and debris, the cell pellets were stained with fluorochrome-conjugated anti-mouse antibodies at a dilution of 1:200. Single-cell suspensions were preincubated with Fc Block antibody (BD Pharmingen) in PBS + 2% heat-inactivated FBS for 10 minutes at room temperature before staining. Cells were incubated for 30 minutes at 4°C with the following antibodies: BUV395 anti-CD8 (clone 53-6.7, BD Biosciences), PE anti-CD44 (clone 1M7, BioLegend), anti-NK1.1 (clone PK136, BioLegend), APC anti-CD103 (clone 2E7; eBioscience, Thermo Fisher Scientific), BV711 anti-CD3 (clone 145-2C11, BioLegend), BV421 anti-CD69 (clone H1.2F3, BioLegend), Alexa Fluor 700 anti-CD45 (clone 30F-11, BioLegend), BV605 anti-CD4 (clone RM4-4, BioLegend), Alexa Fluor 488 anti-F4/80 (clone BM8, BioLegend), APC anti-P2RY12 (clone S16007D, BioLegend), PE-Cy7 anti-Ly6G (clone 1A8, BioLegend), APC-Cy7 anti-CD11c (clone N418, BioLegend), BV711 CD11b (clone M1/70, BioLegend), BV421 anti–I-A/I-E (clone M5/114.15.2, BioLegend), BV605 anti-Ly6C (clone HK1.4, BioLegend), BV750 anti-CD223/Lag3 (clone C9B7W, BD), BV421 anti-CD279/PD-1 (clone RMP1-30, BD), and APC anti-Tim3 (clone RMT3-23, BioLegend). Dead cells were identified with Fixable Viability Dye eFluor 506 (eBioscience, Thermo Fisher Scientific). Cells were stained for 30 minutes at 4°C, washed, and fixed and permeabilized with Foxp3/Transcription Factor Staining Buffer Set (eBioscience, Thermo Fisher Scientific, 00-5523-00), followed by intracellular staining with PE-Cy5 anti-FoxP3 (clone FJK-16s, eBioscience, Thermo Fisher Scientific) and PE anti-Nos2 (clone CXNFT; eBioscience, Thermo Fisher Scientific). Our gating strategy is shown in Supplemental Figure 2. Absolute cell counts were determined using TruCount beads (BD Biosciences). Flow cytometry data were acquired on a cytometer (BDX-20; BD Biosciences) and analyzed using FlowJo software (Tree Star).

### Histology.

Brain tissues were fixed in 10% buffered formalin (Thermo Fisher Scientific), embedded in paraffin, cut into 5 μm thick sections, and stained with hematoxylin and eosin (Thermo Fisher Scientific). Whole-tissue scans at 20× original magnification were obtained on a Zeiss Axio Scan Z1 bright-field/fluorescence Slide Scanner, and images were postprocessed using the Zeiss Zen Blue 3.1 software.

### Statistics.

All data are from at least 2 independent biological experiments (unless mentioned otherwise) with multiple mice in each group. Only animals that survived tumor and/or virus implantation procedures were used for the study. Cohort size and number of technical replicates are specified in each figure legend. Statistical differences were calculated with Prism 8 (GraphPad) using log-rank Mantel-Cox tests (survival), unpaired 2-tailed Mann-Whitney *U* tests (to compare 2 groups with nonparametric data distribution), or 2-way ANOVA with Dunnett’s multiple-comparison test (to compare more than 2 groups with parametric distribution). Differences with a *P* value of less than 0.05 were defined as significant.

### Study approval.

This study was performed in accordance with the recommendations in the *Guide for the Care and Use of Laboratory Animals* of the NIH (National Academies Press, 2011). The protocols were approved by the Institutional Animal Care and Use Committee at the Washington University School of Medicine (assurance A338101). Inoculations were performed under anesthesia induced and maintained with ketamine hydrochloride and xylazine, and all efforts were made to minimize animal suffering.

## Author contributions

SN, LM, AJ, JG, SSB, SS, JNR, PYS, MSD, and MGC designed the studies. TH, LM, AJ, SN, and JG performed tumor implantations and ZIKV treatments. SN, LM, AJ, and TH performed survival analysis. SN and LM performed immune cell processing for flow cytometry and analysis. LM and SN processed images for histology. TH, LM, AJ, SN, SSB, and JG analyzed bioluminescence imaging data. SSB performed neurobehavioral assessments. CRFG, CS, and PYS constructed 10 3′-UTR-ZIKV. JG and SN generated and titered all viral stocks. LM performed the in vitro infections on human and mouse cells. HO provided the SB28 cells. SN compiled the figures with input from AG and MGC. SN, MGC, MSD, and AJ wrote the initial draft, and other authors contributed to the final manuscript. Co–first authorships were assigned after evaluating the percentage contribution to every experiment in this manuscript.

## Supplementary Material

Supplemental data

## Version 1 11/24/2020

In-Press Preview

## Version 2 01/11/2021

Electronic publication
