# Supplementary material for: Zika virus oncolytic activity requires CD8+ T cells and is boosted by immune checkpoint blockade
Source: JCI Insight. 2021 Jan 11;6(1):e144619. doi: 10.1172/jci.insight.144619 (PMC7821591; doi:10.1172/jci.insight.144619)
Supplement: Supplemental data [file jciinsight-6-144619-s088.pdf]

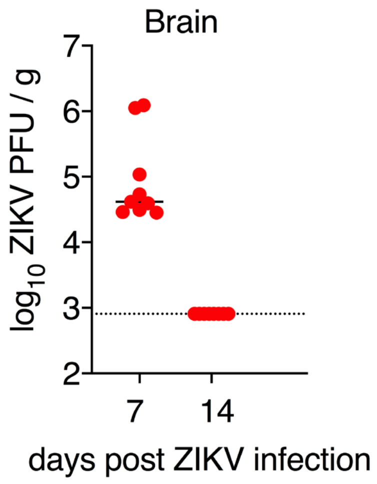

Figure S1. ZIKV infectious viral particles in the brain. Related to Fig 1. Mice were implanted with 4 × 10^4 of GL261 cells and treated with ZIKV on day 7. ZIKV viral particles were quantified by plaque assay at day 14 and day 21 post tumor implantation (or day 7 and day 14 post ZIKV infection). The horizontal lines indicate median values. The dotted line indicates the limit of detection. All data are pooled from at least two independent experiments.

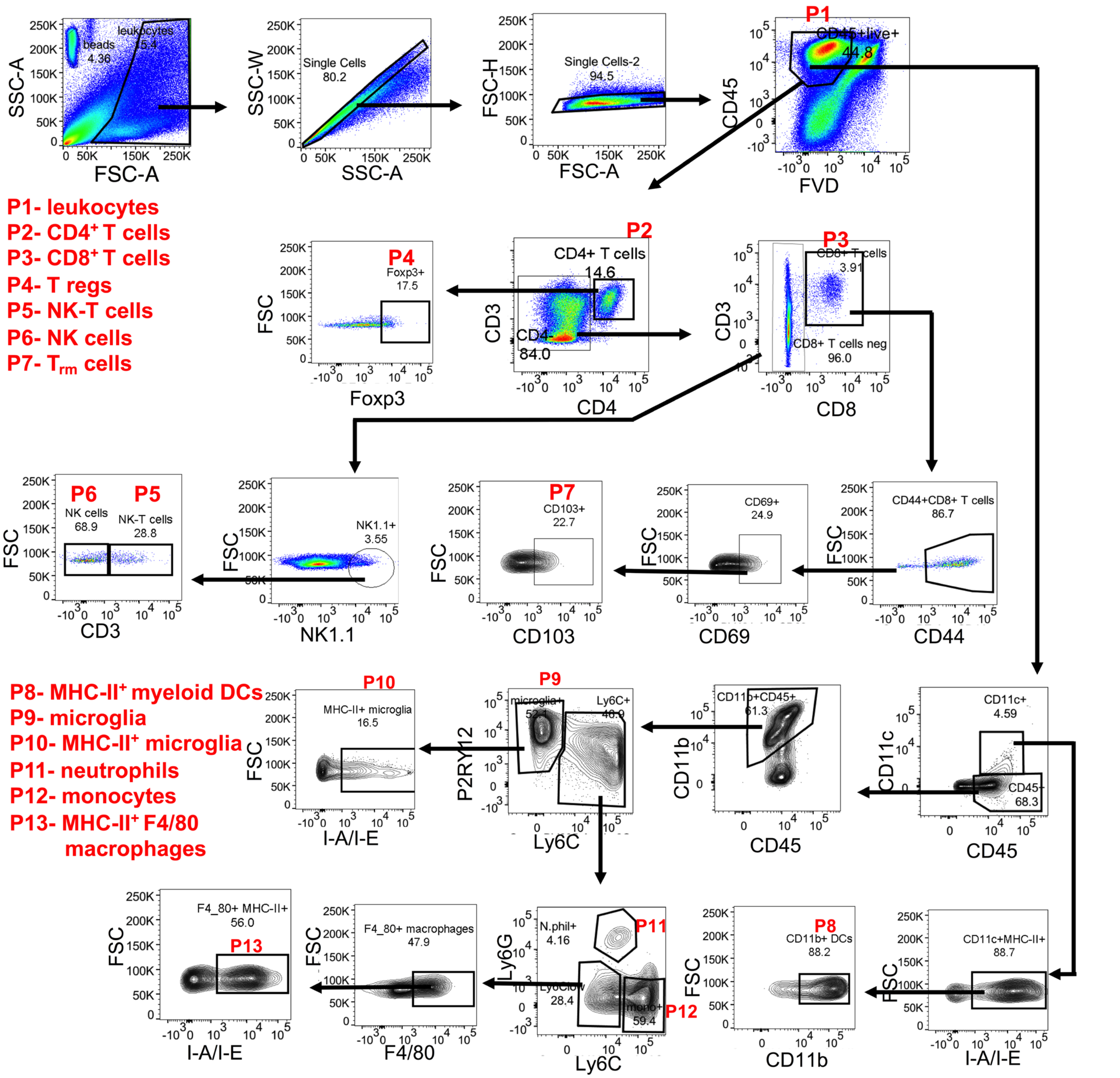

Figure S2. Gating strategy of immune cell infiltrates in the brains of glioma bearing mice following PBS or ZIKV treatments. Related to Figure 2 and Figure 4. Analysis of immune cell populations in the brains of mice bearing glioma, treated with PBS or ZIKV.

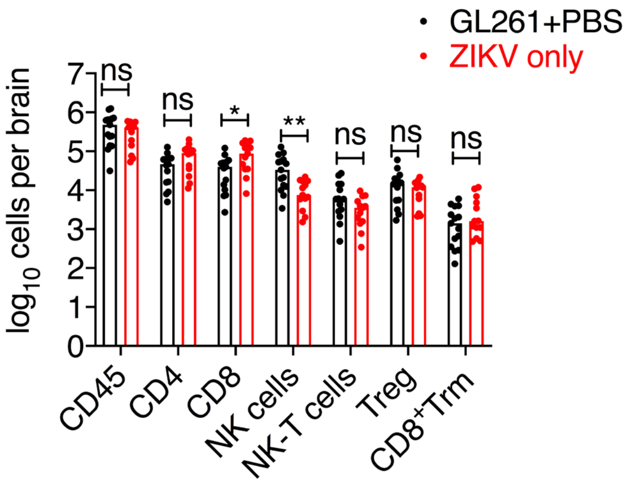

Figure S3. **Absolute numbers of lymphoid cells in GL261 tumor bearing mice.** Related to Figure 2. Mice were implanted with 4 × 10^4 of CT2A cells and treated with PBS on day 7. No-tumor mice (controls) were challenged with 10^5 FFU of mouse-adapted ZIKV Dakar on the same day as PBS treatment in the tumor-bearing mice. Total numbers of immune cells were analyzed from the brain by flow cytometry. Statistical differences were determined by Mann-Whitney test *, $P< 0.05$; **, $P< 0.01$; ns, not significant. All data are pooled from at least two independent experiments.

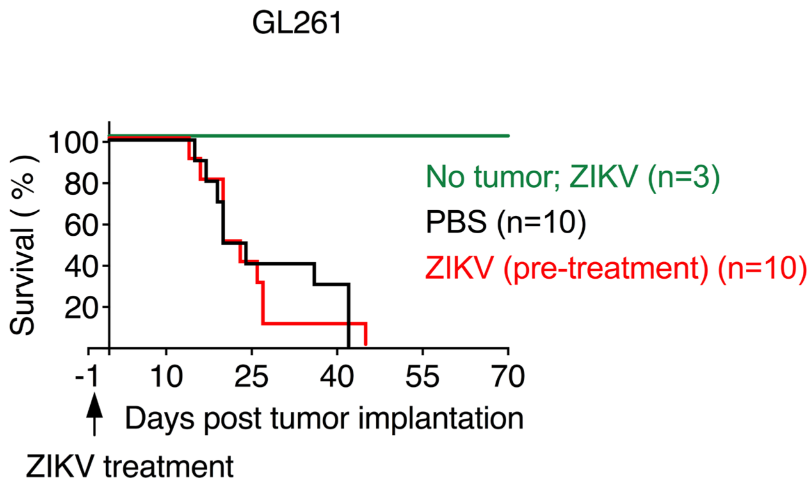

Figure S4. **ZIKV treatment prior to tumor implantation does not improve survival from glioma.** Related to Figure 2. Mice were treated with 10^5 FFU of mouse-adapted ZIKV Dakar (*n*=10) or PBS (*n*=10) prior to being implanted with 4 × 10^4 of CT2A cells. No tumor, mouse-adapted ZIKV Dakar treated mice acted as controls (*n*=3). Data is from one experiment.

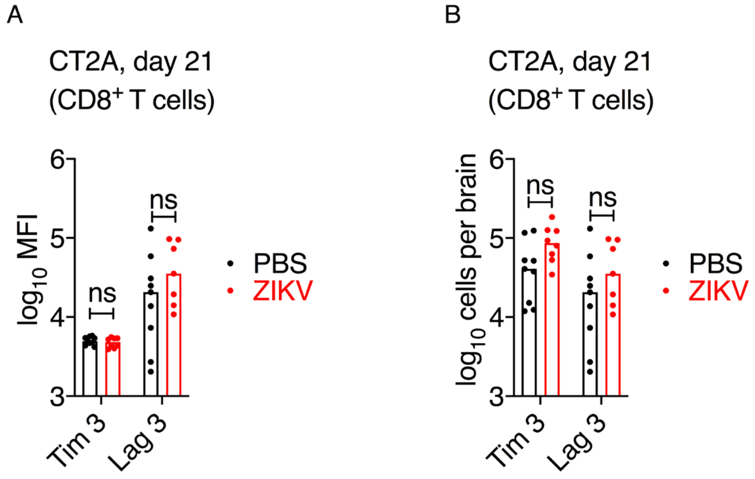

Figure S5. **ZIKV treatment does not impact the expression or absolute numbers of Tim3+ or Lag3+ T cells.** Related to Figure 3**.** Mice were implanted with 4×10^4 of CT2A cells and treated with 10^5 FFU of mouse-adapted ZIKV-Dakar or PBS on day 7. (**A**) Mean fluorescence intensity (MFI) of Tim3 and Lag3 expression in $CD8^+$ T cells from brains of PBS-treated or ZIKV-treated glioma bearing mice. (**B**) Absolute numbers of $Tim3^+$ $CD8^+$ T cells and $Lag3^+CD8^+$ T cells from brains of PBS-treated or ZIKV-treated glioma bearing mice. Bars represent median values. Data are from two independent experiments. Statistical differences were determined by Mann**-**Whitney test (ns, not-significant).

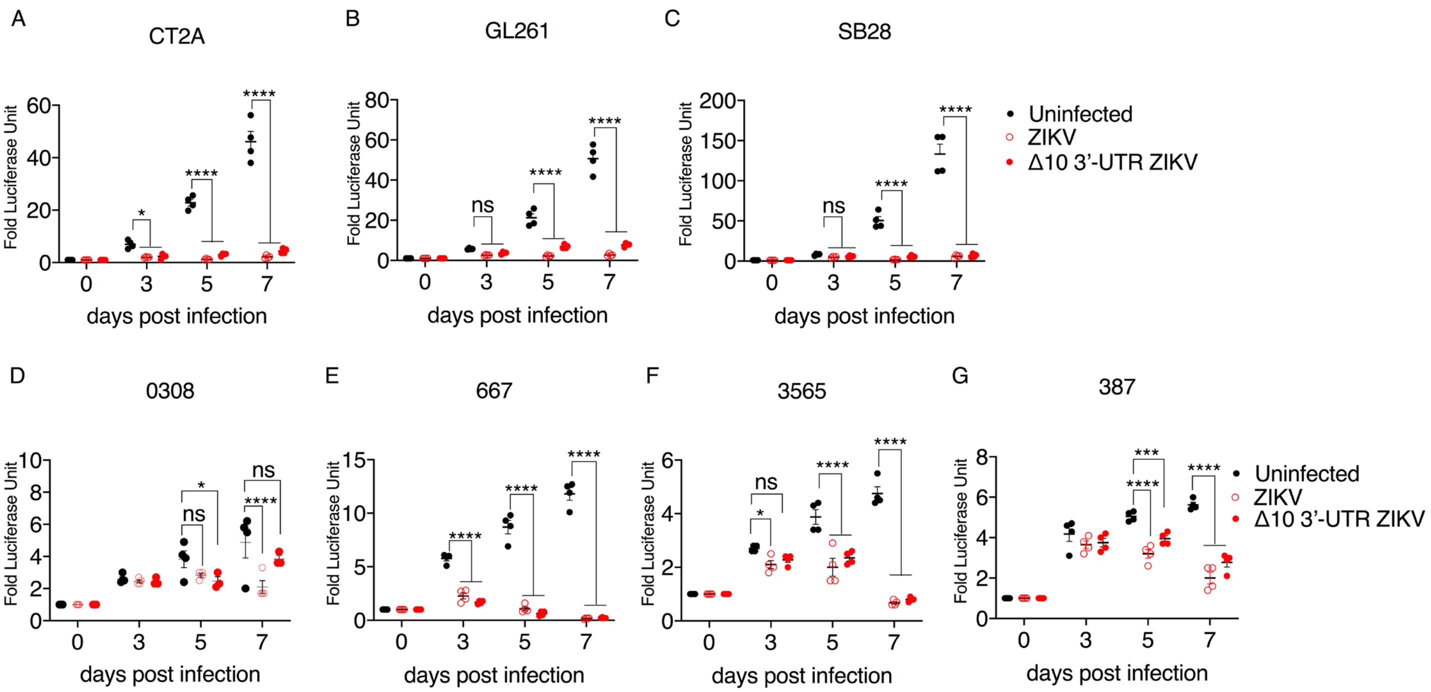

Figure S6. Parental mouse-adapted ZIKV and Δ10 3'-UTR ZIKV inhibit the growth of GSCs. Related to Figure 5. (**A-C**) Mouse (CT2A, GL261 and SB28) and (**D-G**) human GSCs (0308, 667, 3565, 387) cell lines were mock treated or incubated with parental mouse-adapted ZIKV-Dakar or Δ10 3'-UTR ZIKV (MOI of 5) and assayed for relative cell number normalized to day 0 at 3, 5 and 7 days post infection. Statistical differences were determined by two-way ANOVA with Dunnet's post-test. *, $P < 0.05$; **, $P < 0.01$; ***, $P < 0.001$; ****, $P < 0.0001$; ns, not significant. Data are pooled from two independent experiments.
